# A seeded propagation of Cu, Zn-superoxide dismutase aggregates in amyotrophic lateral sclerosis

**DOI:** 10.3389/fncel.2014.00083

**Published:** 2014-03-18

**Authors:** Mariko Ogawa, Yoshiaki Furukawa

**Affiliations:** Laboratory for Mechanistic Chemistry of Biomolecules, Department of Chemistry, Keio UniversityYokohama, Japan

**Keywords:** SOD1, aggregation, seeding reaction, protein misfolding, amyloid

## Abstract

Abnormal accumulation of protein inclusions in motor neurons has been known as a major pathological change in amyotrophic lateral sclerosis (ALS). Increasing numbers of proteins including mutant Cu, Zn-superoxide dismutase (SOD1) have been identified as constituents of pathological inclusions in a form of insoluble fibrillar aggregates. Notably, protein fibrillar aggregates exhibit a self-perpetuating property, which can convert a soluble native protein into insoluble fibrillar aggregates. Such “seeding reaction” of protein fibrils can accelerate the aggregation significantly and would contribute to the spread of inclusion pathologies from an affected cell to its neighboring cells in neurodegenerative diseases. In ALS, a pathological change first occurs at the site of disease onset and then propagates throughout the affected tissues in a time-dependent manner; therefore, it can be assumed that seeded aggregation may be the key factor of disease progression in ALS. In this mini review, we will briefly summarize recent studies on possible roles of a seeded aggregation of SOD1 in pathomechanism of ALS.

## Introduction

Many proteins gain physiological functions by folding into their own unique three-dimensional structures, and any disturbance during this folding process potentially disrupts protein functions, which is considered to cause a variety of diseases (Morimoto, 2008). Among those, neurodegenerative diseases have been well characterized by abnormal accumulation of “mis”-folded proteins in brains and spinal cords of patients (Soto, 2003; Ross and Poirier, 2004). More specifically, certain misfolded proteins form insoluble, fibrillar aggregates that are rich in β-sheet structures, widely known as amyloid (Nelson et al., 2005).

In many neurodegenerative diseases, neurological symptoms appear in middle age (50 years ~), suggesting that it is a rare event for proteins to become misfolded/aggregated. In fact, protein fibrillar aggregation *in vitro* requires a significant conformational conversion of proteins to form oligomers (also known as “nucleus”), which is a rate-limiting step of the overall aggregation reaction (Harper and Lansbury, 1997). Once the nucleus forms, however, it functions as a structural template (or so called “seed”) to convert native proteins into β-sheet-rich structures and then elongate the protein fibril. This mechanism, which accelerates and even triggers protein aggregation, is called the seeding reaction. While it remains controversial whether protein aggregation is the direct cause or a mere result of neurodegeneration (Winklhofer et al., 2008; Treusch et al., 2009), this seeding mechanism may explain why many neurodegenerative diseases progress rapidly after the symptoms first appear.

One notable example for a seeding reaction is the infectivity of Prion diseases, in which the spread of fibrillar aggregates of prion proteins is considered to be the main cause of neurodegeneration (Prusiner, 1982; Aguzzi and Rajendran, 2009). Fibrils of prion proteins are considered to work as infectious agents that can be transmitted between individuals. As exemplified in kuru (Gibbs et al., 1980), eating affected tissues of the disease could introduce fibrillar prion aggregates into a brain of a healthy control as seeds and thereby trigger fibrillation of prion proteins and cause neurodegeneration. While no infectivity between individuals has been reported in neurodegenerative diseases besides prion diseases so far, a seeding phenomenon appears to be common to protein fibrillar aggregates (Dobson, 1999); therefore, increasing numbers of researchers have pursued possible roles of seeding reactions in pathologies of neurodegenerative diseases (Aguzzi and Rajendran, 2009; Polymenidou and Cleveland, 2011; Soto, 2012). For example, Alzheimer’s disease (AD) is characterized by fibrillar aggregation of Aβ peptides in brains (Hardy and Selkoe, 2002), and accelerated accumulation of Aβ fibrils has been confirmed in primate and rodent models that are injected with brain homogenates of an AD patient (Meyer-Luehmann et al., 2006; Ridley et al., 2006). Several other pathogenic proteins also forms fibrillar aggregates *in vitro*, which have been tested for their *in vivo* seeding activity by being transduced into cultured cells and brains of transgenic mouse model (Aguzzi and Rajendran, 2009; Polymenidou and Cleveland, 2011; Soto, 2012). A seeding reaction of protein fibrils is thus considered to play important roles in pathological progression of neurodegenerative diseases, and in this mini review, we will focus upon roles of seeded aggregation of proteins in pathologies of amyotrophic lateral sclerosis (ALS).

## A seeded fibrillation of superoxide dismutase (SOD1) as a pathological propagation of amyotrophic lateral sclerosis (ALS)

ALS is a devastating motor neuron disease, mainly caused by abnormal accumulation of inclusions in the spinal cord (Bruijn et al., 2004). Notably, ALS has been known to occur as a focal process, which spreads contiguously throughout upper and lower motor neurons (Ravits and La Spada, 2009; Holmes and Diamond, 2012; Kanouchi et al., 2012). In other words, motor neuron degeneration in ALS is an orderly and actively propagating process, which appears to share characteristics of a seeded aggregation of proteins seen in Prion diseases.

Most ALS cases (~90%) are sporadic with no known genetic factors (sporadic ALS, sALS), while the remaining cases have been known to exhibit a family history (familial ALS, fALS; Robberecht and Philips, 2013). In 1993, dominant mutations in the gene encoding Cu, Zn-superoxide dismutase (SOD1) were identified as one of major genetic causes of fALS (Rosen et al., 1993), and mutant SOD1 proteins have been known to accumulate abnormally in the form of insoluble inclusions within affected spinal motor neurons of SOD1-related fALS patients (Bruijn et al., 1998). Ultrastructural analysis of SOD1-positive inclusions in fALS cases has identified their fibrillar morphologies (Kato et al., 2000); however, those inclusions were not stained by amyloid-diagnostic dye, Thioflavin S, which has made it controversial whether fibrillar aggregates of mutant SOD1 *in vivo* are rich in β-sheets (Kerman et al., 2010). Moreover, SOD1-positive inclusions have never been isolated from fALS cases, so further biochemical tests will be required to characterize pathological SOD1 aggregates.

In contrast, SOD1-positive inclusions with ALS-like symptoms were reproduced in a fALS-model mouse expressing human SOD1 with a pathogenic mutation (Turner and Talbot, 2008) and were found to be stained by Thioflavin S, supporting the formation of amyloid-like, β-sheet-rich fibrils in mouse (Wang et al., 2002; Furukawa et al., 2008). Insoluble SOD1 aggregates were also successfully isolated from the spinal cords of affected fALS-model mice, and quite notably, those SOD1 aggregates exhibited seeding activity toward fibrillation of purified SOD1 proteins *in vitro*. Chia et al. have prepared homogenates of spinal cords of transgenic mice expressing human SOD1 with G93A mutation and shown that the homogenates triggered fibrillation of wild-type as well as G93A-mutant human SOD1 proteins under *in vitro* conditions with acidic pH of solution in the presence of a chaotropic reagent, guanidine hydrochloride (Chia et al., 2010). While destabilization of SOD1 proteins under artificial conditions appears to be required for a seeded acceleration of fibrillar aggregation, inclusions containing mutant SOD1 would function as seeds and thereby contribute to propagation of pathological changes among contiguous motor neurons and then disease progression of SOD1-related fALS cases.

Fibrillogenic propensities of SOD1 proteins have been well characterized in *in vitro* studies using purified recombinant proteins. SOD1 is a cytoplasmic enzyme (Chang et al., 1988) that catalyzes the conversion of superoxide radicals to hydrogen peroxide and oxygen (McCord and Fridovich, 1969) and is activated by binding of a catalytic copper and a structural zinc ion and also by forming an intramolecular disulfide bond (Furukawa et al., 2004). Wild-type holo-SOD1 with a disulfide bond exhibits high thermostability (*T_m_* 90°C), conferring significant resistance to structural changes and aggregation (Forman and Fridovich, 1973). In contrast, when SOD1 lacks both metal ions and a disulfide bond (apo-SOD1^SH^), its melting temperature decreases down to 43°C and become more prone to misfolding and aggregation at physiological temperature (Furukawa and O’Halloran, 2005). *In vitro* aggregates of human SOD1 polypeptide without any modifications possess amyloid-like characters with fibrillar morphologies and show a seeding activity to accelerate fibrillation of native human SOD1 proteins (Furukawa et al., 2008). More importantly, amyloid-like fibrils of human apo-SOD1^SH^ retain their seeding activity in the intracellular environment; transduction of those human SOD1 fibrils into cultured cells (mouse neuroblastoma, *Neuro2a*) has been shown to trigger the aggregation of stably-transfected human SOD1 (Furukawa et al., 2013).

fALS-causing mutations have been shown to decrease affinity for copper/zinc ions and/or stability of a disulfide bond (Hayward et al., 2002; Tiwari and Hayward, 2003; Furukawa et al., 2008). Therefore, in a reducing environment of the cytoplasm with high metal-chelating capacity, pathogenic mutations are supposed to increase intracellular fractions of fibrillation-prone apo-SOD1^SH^ (Furukawa et al., 2008). Nonetheless, it remains unclear how mutant SOD1 forms aggregates under pathological conditions. To elucidate how SOD1 aggregates form, several pathways for aggregation have been proposed in SOD1 proteins *in vitro* (Furukawa, 2012b). As reported by Munch et al. mutant SOD1 was found to form fibrillo-granular aggregates by addition of trifluoroethanol (TFE; Münch and Bertolotti, 2010), which penetrated inside neuronal cells through macropinocytosis and then acted as seeds to trigger intracellular aggregation of endogenously expressed SOD1 variants (Münch et al., 2011). Once SOD1 aggregation occurs in a cell, the aggregates can be released to the extracellular space and then transferred from cell to cell (*vide infra*). Intracellular aggregation of mutant SOD1 is thus considered to be persistent and heritable after passages, supporting prion-like propagation of aggregation phenotypes.

As mentioned above, two distinct types of SOD1 aggregates, i.e., apo-SOD1^SH^ amyloids and TFE-induced aggregates, have been found to function as seeds to trigger SOD1 aggregation intracellularly, but their structural and biochemical properties depend on how aggregation was induced (Furukawa et al., 2008; Münch and Bertolotti, 2010). Based upon previous *in vitro* studies, several distinct pathways for aggregation are possible in SOD1 (Toichi et al., 2013) and are expected to produce SOD1 aggregates with a varying degree of a seeding activity. This might describe heterologous progression and severity of diseases among SOD1-related fALS patients. Indeed, disease phenotypes of fALS cases have been known to be variable among different mutations in SOD1 (Wang et al., 2008), and furthermore, mutation-dependent structures of SOD1 fibrils closely correlate with their distinct biochemical properties (Furukawa et al., 2010). Therefore, it will be interesting to test if SOD1 fibrils with different mutations exhibit distinct activity as seeds *in vitro* and *in vivo*.

## Propagation of protein misfolding in amyotrophic lateral sclerosis (ALS)

In a seeding reaction, sheared pieces of insoluble fibrils can act as structural templates for a “phase-like transition” from soluble native conformers to generally insoluble fibrillar state, but this view now appears to be necessary for revision. Grad et al. utilized antibodies (3H1 and 10C12) that exclusively recognize misfolded SOD1 with disease-specific epitopes, which are not available in the natively folded state, and showed that misfolding of endogenous wild-type SOD1 in human cells (e.g., human embryonic kidney 293 cells (HEK293)) is induced by co-expression of a soluble misfolded form of human SOD1 with pathogenic mutations (Grad et al., 2011). In other words, soluble misfolded conformers of SOD1 are also transmissible without adopting classical, insoluble fibrillar states. Furthermore, transient expression of mutant human SOD1 in murine cells (e.g*., Neuro2a*) did not induce misfolding of endogenous mouse wild-type SOD1 (Grad et al., 2011). The difference lies in the amino acid sequence of murine and human SOD1, where the only tryptophan in human SOD1 (Trp32) is replaced by serine in murine counterpart. Indeed, misfolding of wild-type human SOD1 was observed by human SOD1 with a pathogenic (G127X) mutation but was significantly mitigated when G127X human SOD1 with W32S mutation was used (Grad et al., 2011). Trp32 in human SOD1 is highly solvent-exposed and distant from the native dimer interface, which might provide an alternative site for abnormal intermolecular interactions through hydrophobic interactions. It is interesting to note that expression of mutant TAR DNA binding protein 43 (TDP-43) and Fused in Sarcoma (FUS), pathogenic proteins also known to be found in ALS patients (Arai et al., 2006; Neumann et al., 2006; Kwiatkowski et al., 2009; Vance et al., 2009), can increase the immunoreactivity for misfolded SOD1 using a disease-specific antibody (3H1), both in patients and cultured human cells (SH-SY5Y; Pokrishevsky et al., 2012). While pathological involvement of wild-type SOD1 in ALS remains to be established, aberrant conformers of wild-type SOD1 have been reported in sporadic ALS with no genetic background (Furukawa, 2012a). Accordingly, toxic conformers of SOD1 might be produced by abnormal interactions of folded SOD1 with misfolded SOD1 or other proteins (such as TDP-43/FUS) at the site surrounding Trp32. In other words, as proposed in the template-assisted misfolding of prion proteins (Horwich and Weissman, 1997), soluble but misfolded conformers of protein molecules can be propagated through abnormal interactions among homologous proteins even without the formation of classical amyloid-like fibrils.

## A cell-to-cell transfer of intracellular superoxide dismutase (SOD1)

SOD1 is known as one of major intracellular proteins, and most of SOD1 (~70%) exist in the cytoplasm (Chang et al., 1988). To confirm that seeded aggregation or misfolding of SOD1 is the key molecular mechanism of pathological propagation of SOD1-fALS, it is required to understand how intracellular misfolded/aggregated SOD1 is transferred from the cytoplasm to extracellular environment. As a relatively simple process, misfolded/aggregated SOD1 would be released to extracellular environment by death of an affected cell and then phagocytosed by the other cell. Recent studies have nonetheless suggested more sophisticated processes for a cell-to-cell transfer of SOD1 proteins (Grad et al., 2011; Münch et al., 2011). In fact, active secretion of SOD1 to extracellular space has been suggested in several different types of cultured cells (Mondola et al., 1996, 1998), and both wild-type and mutant SOD1 can be also detected in the cerebrospinal fluid of healthy controls as well as fALS patients (Zetterström et al., 2011).

In conditioned media of mouse motor neuron-like hybrid (NSC-34) cell line, impaired secretion of mutant SOD1 was associated with intracellular formation of inclusions and toxicity, suggesting secretion of mutant SOD1 as a beneficial process for cell survival (Turner et al., 2005). In contrast, Urushitani et al. have found that mutant SOD1 proteins are secreted in association with chromogranins and cause microgliosis and neuron death (Urushitani et al., 2006), leading to the idea that suppression of extracellular mutant SOD1 is a promising strategy for therapeutics of SOD1-related fALS cases. Indeed, passive as well as active immunizations targeting extracellular mutant SOD1 proteins have successfully prolonged lifespan of transgenic mice expressing mutant human SOD1 (Urushitani et al., 2007). Toxic roles of secreted SOD1 are further supported by the findings that motor neurons are killed by being co-cultured with astrocytes derived from adult neural progenitor cells isolated from post-mortem lumber spinal cord tissues from sporadic ALS as well as SOD1-related fALS (Haidet-Phillips et al., 2011). Also importantly, suppression of SOD1 in both fALS and sporadic ALS astrocytes was found to negate such toxicity of astrocytes toward motor neurons. Recently, furthermore, Basso et al. have shown the increased release of exosomes from astrocytes overexpressing fALS-causing mutant SOD1 and found that astrocyte-derived exosomes contained mutant SOD1 proteins and were transferred to the cytoplasm of spinal neurons (Basso et al., 2013).

Based upon these results, secretion of SOD1 is considered to occur through several distinct pathways and appears to be a normal physiological process. Experimental evidences are further required to show that secretory vesicles act as a messenger to generate seeding activity of SOD1. More specifically, conformational analysis of SOD1 (folded, misfolded, or fibrillized) included in those vesicles will reveal the molecular mechanism of pathological propagation in ALS through a seeding reaction.

## Summary

As briefly summarized above, increasing numbers of recent studies have supported the idea that misfolding/aggregation of mutant SOD1 is transmissible through a seeding mechanism inside the cell and among cells. In that sense, it is interesting to test pathological roles, if any, of SOD3, which resides at the extracellular matrix and possesses a structural domain almost homologous to SOD1 (Folz and Crapo, 1994). A SOD1-like domain of SOD3 has been shown to exhibit propensities for aggregation (Son et al., 2003), implying its involvement in the formation of seeds that can be taken up by cells. In summary, a seeded aggregation of SOD1 proteins including wild-type SOD1 will be a key event to understand progression/propagation of pathological changes in SOD1-related fALS and even sALS cases without mutations in SOD1, and extracellular SOD1 with aberrant conformations is a promising target for therapeutics of those devastating diseases.

## Conflict of interest statement

The authors declare that the research was conducted in the absence of any commercial or financial relationships that could be construed as a potential conflict of interest.
